# A Conceptual Approach towards Improving Monitoring of Living Conditions for Populations Affected by Desertification, Land Degradation, and Drought

**DOI:** 10.3390/su15129400

**Published:** 2023-06-12

**Authors:** David López-Carr, Narcisa G. Pricope, Kevin M. Mwenda, Gabriel Antunes Daldegan, Alex Zvoleff

**Affiliations:** 1 Department of Geography, University of California, Santa Barbara, CA 94607, USA; 2 Department of Earth and Ocean Sciences, University of North Carolina, Wilmington, NC 28403, USA;; 3 Population Studies and Training Center, Brown University, Providence, RI 02912, USA;; 4 Conservation International, Arlington, VA 22202, USA

**Keywords:** sustainable development goals, desertification, land degradation, and drought (DLDD), UNCCD Strategic Objectives

## Abstract

Addressing the global challenges of desertification, land degradation, and drought (DLDD), and their impacts on achieving sustainable development goals for coupled human-environmental systems is a key component of the 2030 Agenda for Sustainable Development. In particular, Sustainable Development Goal (SDG) 15.3 aims to, “*by 2030, combat desertification, restore degraded land and soil, including land affected by desertification, drought and floods, and strive to achieve a land degradation-neutral world”*. Addressing this challenge is essential for improving the livelihoods of those most affected by DLDD and for safeguarding against the most extreme effects of climate change. This paper introduces a conceptual framework for improved monitoring of DLDD in the context of United Nations Convention to Combat Desertification (UNCCD) Strategic Objective 2 (SO2) and its expected impacts: food security and adequate access to water for people in affected areas are improved; the livelihoods of people in affected areas are improved and diversified; local people, especially women and youth, are empowered and participate in decision-making processes in combating DLDD; and migration forced by desertification and land degradation is substantially reduced. While it is critical to develop methods and tools for assessing DLDD, work is needed first to provide a conceptual roadmap of the human dimensions of vulnerability in relation to DLDD, especially when attempting to create a globally standardized monitoring approach.

## 1. Introduction

### Background and Significance of the UNCCD and SDGs

This paper develops an approach for understanding human and ecological vulnerability and resilience to desertification, land degradation, and drought (DLDD). The conceptual approach developed here can be adapted for other cognate large monitoring processes. Here we focus on the example of supporting Strategic Object 2 (SO2), “*to improve the living conditions of affected populations”*, of the United Nations Convention to Combat Desertification (UNCCD) Strategic Framework for 2018–2030 (Decision 7/COP.13) [1]. SO2 and its four expected impacts are the following:

#### Strategic Objective 2: To improve the living conditions of affected populations

Expected impact 2.1 Food security and adequate access to water for people in affected areas is improved.
Expected impact 2.2 The livelihoods of people in affected areas are improved and diversified.
Expected impact 2.3 Local people, especially women and youth, are empowered and participate in decision-making processes in combating DLDD.
Expected impact 2.4 Migration forced by desertification and land degradation is substantially reduced.

The COP.13 Strategic Framework acknowledges the global challenges of DLDD, and its contributions to “economic, social, and environmental problems” that “pose serious challenges to sustainable development”. It notes that addressing DLDD will involve long-term integrated strategies that simultaneously focus on the improved productivity of land and the rehabilitation, conservation, and sustainable management of land and water resources. The vision of the Strategic Framework is:

“*A future that avoids, minimizes, and reverses desertification/land degradation and mitigates the effects of drought in affected areas at all levels and strives to achieve a land degradation-neutral world consistent with the 2030 Agenda for Sustainable Development, within the scope of the Convention*

(Decision 7/COP.13).”

The conceptual framework presented here builds on Strategic Objective 1 (SO 1) as a presumed baseline, the UNCCD Strategic Objective most closely linked to SDG Target 15.3–Land Degradation Neutrality (LDN), which sets out to *“by 2030, combat desertification, restore degraded land and soil, including land affected by desertification, drought and floods, and strive to achieve a land degradation-neutral world”*. Additionally, SO1 aims to *“improve the condition of affected ecosystems, combat desertification/land degradation, promote sustainable land management and contribute to land degradation neutrality* [2]”.

Land degradation neutrality is an increasingly important problem. There has been no greater impact on the earth’s surface than through deforestation for agricultural and pastoral expansion [3]. Between 2015 and 2020, deforestation claimed an estimated 10 million hectares per year, equivalent to deforesting an area the size of Cuba each year [4]. However, our knowledge about tropical deforestation remains limited by a lack of cohesion between thousands of case studies at the micro scale and gross estimates of forest change at the regional scale. Direct drivers of farm forest conversion can be considered demographic, political–economic, socioeconomic, and ecological. Disciplinary silos have meant that these diverse factors are rarely integrated in holistic research examining deforestation at local and sub-national scales.

For successful and timely monitoring and evaluation of country Party progress toward SDG goals and cognate UNCCD Objectives, it is critical to develop methods and analytical tools for assessing DLDD using free and open geospatial data and platforms for understanding the socio-economic conditions of vulnerable communities in affected areas. However, more work is needed first to better understand the conceptual linkages between human dimensions of vulnerability (and resilience) and DLDD, especially when attempting to create a globally standardized monitoring approach. This paper develops a framework for human population vulnerability and resilience to DLDD.

## 2. Towards Conceptual Integration

An interdisciplinary approach based on a human-natural systems framework is predicated on the empowerment of women and youth, safe and equitable access to food and water, and sustainable livelihoods systems that can moderate migration flows and overall resilience. Some basic principles must be established before developing our conceptual framework. First, drivers of DLDD are insufficiently addressed by any one discipline. Rather, to fully comprehend DLDD and its consequences, demographic, political–economic, socioeconomic, and ecological processes must be examined by experts across these diverse disciplines. Second, for food security and adequate access to water for people in affected areas to be improved, both human and natural systems must be addressed. Third, for the livelihoods of people in affected areas to be improved and diversified, women and youth must be empowered, and the resilience of both social and ecological systems enhanced. Fourth, for local people, especially women and youth, to be optimally empowered and participate in decision-making processes in combating DLDD, food security and access to water security, and the livelihoods of women and youth must be a priority. Lastly, for migration to be substantially reduced, improving resilience to desertification and land degradation is critical.

**Figure 1.**
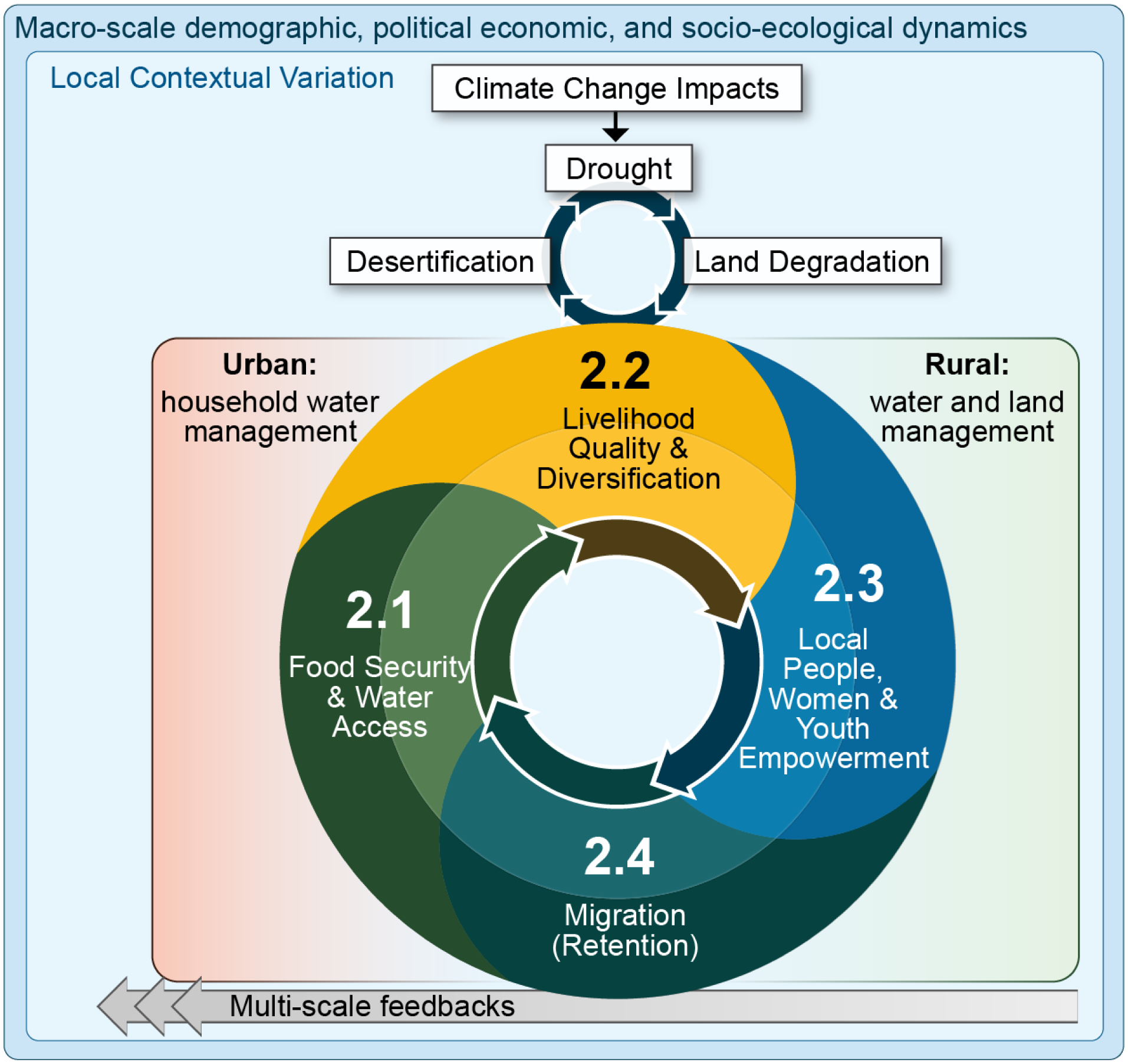
SO2 Community (blue) and ecosystem (green) vulnerability and resilience to DLDD. The four sub-objectives are linked in a cycle. Synergistic “virtuous” cycles empower local people, women, and youth, improve food security, water access, and livelihood quality and diversity while decreasing migration.

Vulnerability is generally considered a function of human exposure to a stressor, an effect (also termed sensitivity or potential impact) and the recovery potential to that stressor (also termed resilience or the capacity to cope with or adapt to slow or fast-onset changes) [5,6]. In the context of DLDD, the vulnerability of human beings and their livelihoods is integral. Livelihoods are intimately linked to DLDD and can have positive and/or negative consequences on DLDD. This is especially the case with migration, where a move may have net positive impacts in one location and potentially net negative outcomes in the other location (or vice versa). As shown in Figure 1, livelihoods connect to DLDD most intimately in developing and rural regions where a large percentage of the population depends on local natural resources. In such instances, livelihood decisions have a direct impact on the environment and thus on LDN efforts. Conversely, the developed world and urban populations often have a greater impact on DLDD secondarily, vis a vis the impact of their consumption, often in remote locations.

Following Figure 1, SO2 sub-objectives are coupled in complementary synergies (more resilience) or, conversely, in mutually deleterious processes (more vulnerability). Households will respond to DLDD in one or multiple ways simultaneously or sequentially. Responses can be to components of demographic, political, socio-economic, and environmental processes at global, national, and local scales, and/or household and individual scales [7–11]. Households and individuals facing DLDD-related resource scarcity may respond economically by changing their livelihood strategy and/or they may respond demographically, e.g., by delaying one or more births or by out-migration (domestically or internationally, temporarily or permanently, among select household members or the entire household) [8–12]. Changing food consumption patterns, water conservation, land use, labor, capital investments, and fertility are common first-line DLDD adaptation responses that can occur within the context of other stressors sequentially or simultaneously by one (or more) household member. Once in situ options have become exhausted (e.g., water and land management and/or off-farm labor, and fertility changes), adaptation strategies may include the decision for a household member or the entire household to out-migrate. Once a decision, or series of decisions is made, other responses ensue, and the household once again is faced with external structures and processes that shape subsequent decision-making. Wherever they are located, the agency of individuals and households to make multiple sequential and simultaneous decisions in response to DLDD will unfold within political-economic structures and environmental processes at multiple scales [12]. To the extent these linkages are synergistic, the empowerment of locals, women, and children can be facilitated. In turn, to the extent locals, especially women and children, are more empowered in decision-making regarding their livelihoods and use of natural resources, the more likely livelihoods, food and water security, and migration retention are enhanced.

When dealing with the human dimensions of DLDD, the appropriate scale of analysis is no longer the ecosystem, as in SO1, but rather the anthropomorphic: individual, household, and community (as shown in Figure 1). In sum, SO2′s four sub-objectives are cyclically connected. Synergistic “virtuous” cycles empower local people, women, and youth, improve food security, water access, and livelihood quality and diversity, while decreasing migration. Conversely, “vicious” cycles unfold when the converse occurs. In the following sections, we discuss how we conceptually operationalize each of these four expected SO2 impacts.

### 2.1. Expected Impact 2.1: Food Security and Water Access

Food security is a critical dimension of the health and wellbeing of households and communities. Sustainable Development Goal 2, *to reduce hunger by 2030 to zero*, is unlikely to be achieved in this timeframe given that the number of people who suffer from food insecurity (defined as the *disruption of food intake or eating patterns because of lack of money and other resources*) and hunger has begun to slowly increase since 2015 to reach 750 million people in 2019 (United Nations SDGs https://www.un.org/sustainabledevelopment/hunger/ accessed on 1 May 2023). Reduced food security, malnutrition, and hunger cause diverse negative outcomes, both in children and adults, and may pose severe deleterious effects on the long-term health of affected populations, even with short-term exposure. Hunger is commonly measured by the prevalence of undernourishment–SDG Indicator 2.1.1; where undernourishment means that a person is not able to acquire enough food to meet the daily minimum dietary energy requirements over a period of one year.

**Figure 2.**
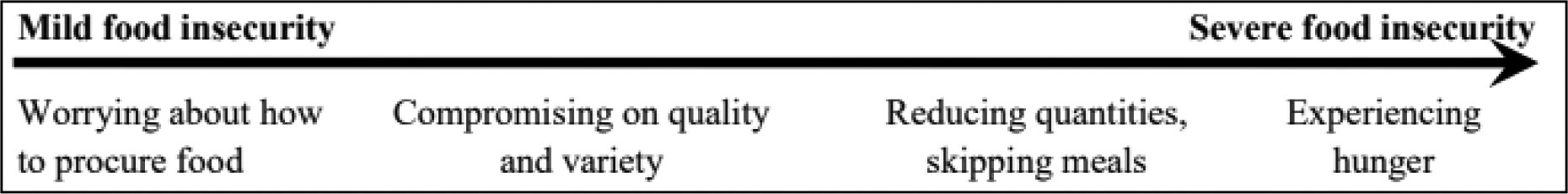
Food insecurity severity along a continuous scale. Source: Food and Agricultural Organization.

The United Nations (UN) Food and Agricultural Organization (FAO) defines hunger as being synonymous with chronic undernourishment. The FAO conceptualizes food insecurity along a continuum (Figure 2) based on the Food Insecurity Experience Scale http://www.fao.org/in-action/voices-of-the-hungry/fies/en/ accessed on 1 May 2023 (FIES; also, SDG Indicator 2.1.2). The FIES is a quantitative metric of severity of food insecurity at the household or individual level that relies on people’s direct yes/no responses to eight brief questions regarding their access to adequate food http://www.fao.org/3/a-i7835e.pdf accessed on 1 May 2023. It is a statistical measurement scale like other widely accepted statistical scales designed to measure unobservable traits such as aptitude/intelligence, personality, and a broad range of social, psychological and health-related conditions. Most relevant to national and global monitoring of food insecurity are the moderate and severe food insecurity categories. Moderate food insecurity describes people facing uncertainties about their ability to obtain food and having been forced to reduce, at times, the quantity and/or quality of food due to lack of money or other resources. Severe food insecurity refers to people who are likely to run out of food, have experienced hunger, and, at the most extreme, have gone for days without eating, putting their health and wellbeing at grave risk. Since SDG Indicator 2.1.2 refers to the total number of people suffering from food insecurity, even at moderate levels, the number will be higher than those suffering from hunger. Since an indicator for food insecurity or hunger has not been identified in the UNCCD Strategic Framework, we propose the use of the FIES. Water access could follow a similar continuum from mild to severe water access deficit.

Following Figure 1, food security and water access operate synergistically with the other three expected outcomes. When livelihood quality and diversification is enhanced, food security and water access are often increased. Similarly, the empowerment of locals, women, and youth can help catalyze empowerment surrounding food security and water access. Lastly, to the extent that food security and water access are sustainable, the need to migrate may decrease, increasing migrant retention. Of course, food security and water access will manifest differently in rural and urban settings. In rural areas, household land and water management will be intimately linked to natural resource management, while in urban areas, enhanced livelihoods, infrastructure, functioning markets, and political transparency and will all will converge to impact food and water security.

### 2.2. Expected Impact 2.2: Livelihoods Quality and Diversification

Socially and economically marginalized populations, communities, and households tend to be disproportionally vulnerable to climate change and the combined effects of DLDD. The ability to identify and quantify the makeup, distribution, and relative vulnerability of such populations, communities, and households is critical in reinforcing livelihood resilience in order to enhance positive adaptations to DLDD [13,14]. Livelihoods encompass how people, either individually at the household level or collectively at the community level, obtain the necessary resources for survival, and their respective capacities and ways of living. Major factors that combine to make up a livelihood include food, income, and assets. Environmentally sustainable livelihoods maintain or enhance the assets on which livelihoods depend at various scales. Livelihoods are socially sustainable when they successfully adapt to stresses and shocks, such that they can continue to provide for future generations.

**Figure 3.**
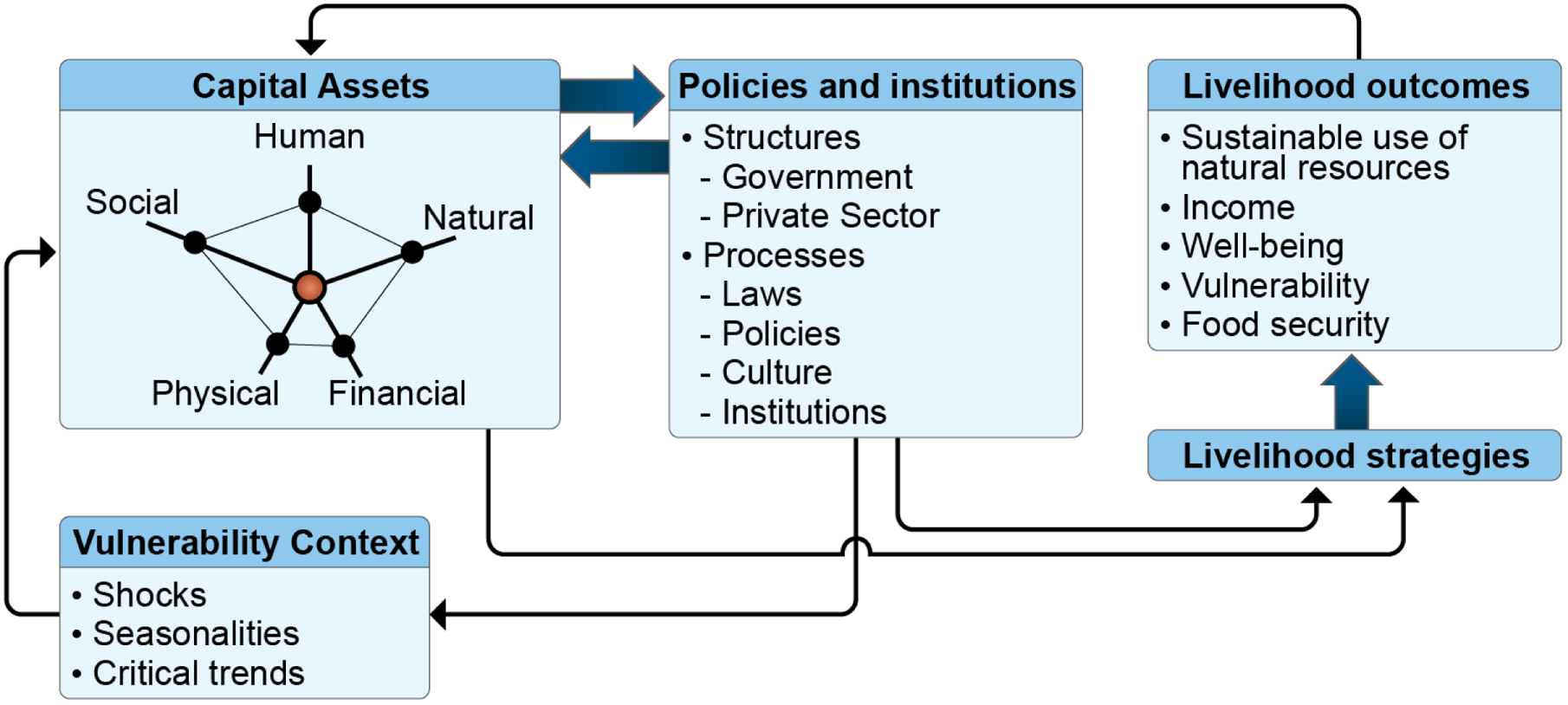
The sustainable livelihoods approach improves understandings of the livelihoods of the poor and relevant outcomes for populations and ecosystems. Adapted from Serrat [16], with credit to the Asian Development Bank (http://creativecommons.org/licenses/by-nc/3.0/igo/, accessed on 1 April 2023).

A livelihoods framework characterizes households as making decisions regarding livelihood activities based on available natural, social, human, physical, and financial capital. The examination of different types of capital allows for a more complete understanding of population, poverty, and environment relationships. Pertinent to assessing DLDD, de Sherbinin et al. [15] have demonstrated that a livelihoods framework can be applied to assess a vicious circle model (VCM) of population, poverty, environment, and climate dynamics. According to the VCM, positive feedbacks at the household level among population growth, poverty, and environmental degradation lead to a downward spiral for poor households. Similarly, a sustainable livelihoods approach improves understanding of the livelihoods of the poor (Figure 3). It organizes the factors that constrain or enhance livelihood opportunities and show relationships among them. This approach helps to plan development activities and to assess the contribution that existing activities have made to sustaining livelihoods. According to this model, livelihood outcomes are impacted by human, social, physical, financial, and natural capital assets, and by structural and procedural policies and institutions. In turn, these policies and institutions impact the vulnerability context which underlies the capital assets. Within this holistic framework, livelihood outcomes impact capital assets and the causal loop begins anew.

In a briefing note published by the UNCCD, data gathered from over 800 sub-national regions demonstrated that places with the highest proportion of degraded land have the most adverse socio-economic performance, both in terms of high poverty rates and high levels of income inequality [17]. Linkages can also be explored in the reverse direction, which is how poverty, income, and wealth can induce land degradation under certain circumstances. The UNCCD concluded that investing in LDN acts to reduce poverty and inequalities. Thus, monitoring approaches should prioritize poverty and/or inequality in areas affected by DLDD.

As per Figure 1, improved livelihoods can be both a cause and a consequence of improved food security and water access, local, youth, and women’s empowerment, and migrant retention. Improved food and water security allow for human capital to be invested in improved livelihoods, while also providing the nutrition and water necessary for optimal livelihood investments. Similarly, improved livelihoods help households enhance food and water security through increased capital accumulation that can be used to purchase food and water. Local, youth, and women’s empowerment can help accelerate local development of small businesses which will improve livelihoods; improved livelihoods, in turn, help facilitate local, youth, and women’s empowerment. Lastly, the retention of human capital when potential migrants remain in the household means more human capital can be invested in local livelihood improvement; conversely, remittances sent from migrants can also help improve local livelihoods.

### 2.3. Expected Impact 2.3: Local People, Women, and Youth Empowerment

The UNCCD recognizes that supporting women’s rights is imperative to combat DLDD https://www.unccd.int/land-and-life/gender/news-stories?page=1 accessed on 1 May 2023. Similarly, engaging locals and the development of youth-related projects are considered critical for improving sustainable livelihoods consistent with reversing DLDD https://www.unccd.int/sites/default/files/2022-03/UNCCD%20GLO%20WP%20youth.pdf accessed on 1 May 2023. Several factors are crucial for local people, especially women and youth to participate in decision-making processes to combat DLDD. As put forth in the livelihoods framework, the empowerment of locals, especially women and youth, is dependent on several processes. This is where the human capital aspect of the capital assets is crucial. Here we can substitute livelihood outcomes for local, women, and youth empowerment. Indeed, empowerment can be considered a sub-component of a broader definition of livelihoods. Empowerment in decision-making, like other livelihood outcomes, is impacted by human, social, physical, financial, and natural capital assets, as well as by the structures and processes of policies and institutions. In turn, these policies and institutions impact the empowerment context which underlies the capital assets, especially human capital assets. Following the livelihood’s framework, empowerment outcomes also ultimately impact capital assets forming a potentially virtuous cycle.

**Figure 4.**
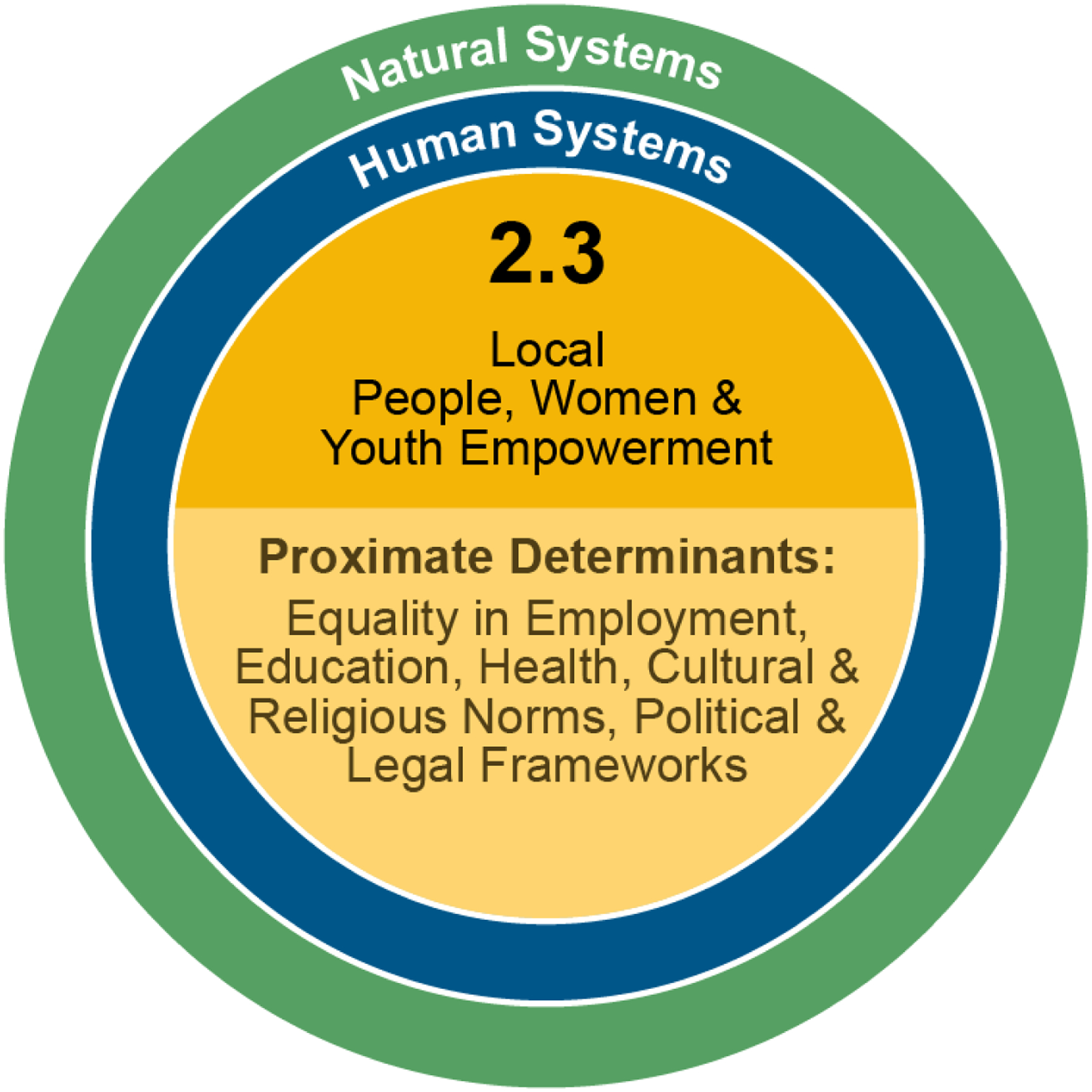
Expected impact 2.3, Local People, Women, and Youth Empowerment. vulnerability and resilience to DLDD are embedded within natural and human systems and ultimately determined by proximate factors that empower local people, women, and youth.

Returning to our conceptual framework in Figure 1, synergistic “virtuous” cycles link together the empowerment of local people, women, and youth with improved food security, water access, and livelihood quality and diversity, while decreasing migration. Locals, women, and youth with improved empowerment to participate in decision-making will have more say in and, therefore, likely more preferable outcomes for how food is made more secure through, e.g., improved cropping techniques, how water access is improved through, e.g., modern filtration devices, each of which improves livelihoods and would act to decrease migration and increase potential migrant retention. Following Figure 4, vulnerability and resilience to DLDD are embedded within natural and human systems and ultimately determined by proximate factors that empower local people, women, and youth. Some of these proximate determinants may include equality in employment, education, health, cultural and religious norms, and political and legal frameworks.

### 2.4. Expected Impact 2.4: Migration (Retention)

Migration data is particularly scarce, especially in the developing world. Migration data linked to DLDD data and the other expected SO2 outcomes are even more rare. Therefore, migration is a good example to highlight the importance of context to understand the potential attribution of DLDD, and the relative effects on ecosystems and people. For example, if population change data suggest net out-migration from a region in which vegetation index-derived landscape greenness had decreased in immediate prior or parallel years, it could be inferred that DLDD may have played a role in pressuring livelihood sustainability for local populations, ultimately pushing people off the land to migrate elsewhere (see, e.g., [18,19]). However, that signal alone may not be enough to determine attribution. Other factors would provide more information suggestive of causation, including trends derived from drought indices, and an on-the-ground understanding of socio-political dynamics, livelihoods, and land and water management. For example, if a war had broken out during the same period of the above hypothetical analysis, fleeing violence could be the main driver of out-migration. Conversely, if the primary livelihoods of residents in the region of interest are white collar, service, or industrial, then political-economic factors might be more important drivers of out-migration than DLDD. If, on the other hand, the primary livelihoods are agro-pastoral, with few wage labor opportunities, DLDD would be a more likely driver. However, even in this case, more information would be useful to convincingly suggest attribution. What adaptations, if any, were possible or observed in the region? Did large landholders consolidate area land and intensify production? If so, to what extent were agro-pastoralists absorbed (if at all) into the changing land regimes as agricultural laborers? If intensification was technologically and capital intensive, we may infer a higher level of out-migration from the region than if DLDD adaptations were labor intensive.

Lastly, most of the world’s population is now urban and the remaining rural areas are rapidly losing population to urban settings. How does migration to an urban destination differ in impact from migration to a rural destination [20]? As a conceptual guide, we can return to the livelihoods approach in which improvements in the various livelihood components will likely reduce out-migration and retain potential migrants. What happens once migrants have moved to their new location in terms of the impact on DLDD can be once again framed by the conceptual models presented here. No measure currently exists that provides a perfect, direct relationship among all the complex components that comprise human and ecosystem vulnerability and resilience. Context is critical, and the critical contextual questions posed above are of the sort that must be pursued when assessing the relative attribution of human responses to change (or stasis) in ecosystem variability and vulnerability. Nevertheless, several concluding points and implications for future research emerge.

**Figure 5.**
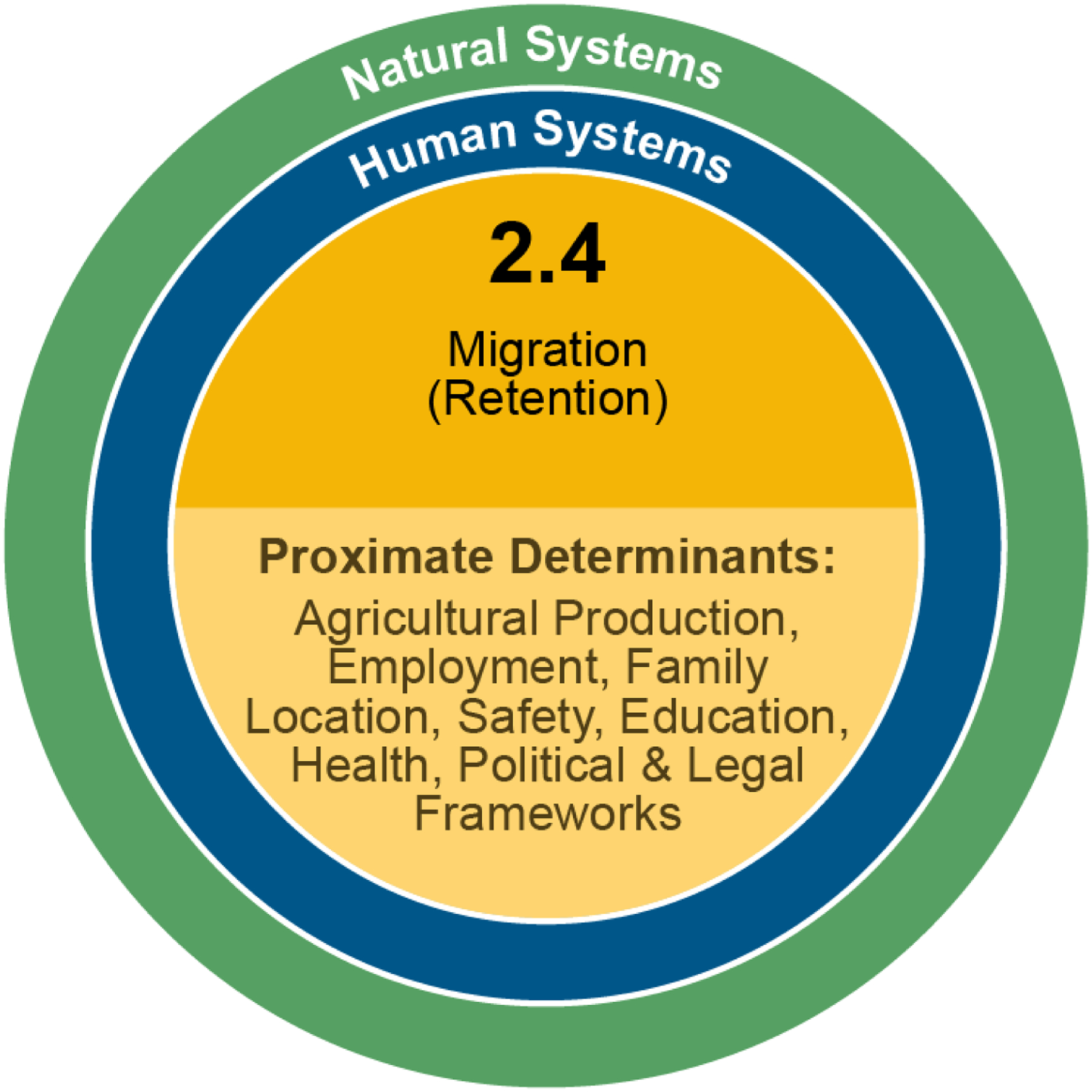
Expected impact 2.4: Migration. Vulnerability and resilience to DLDD are embedded within natural and human systems that ultimately produce proximate determinants that either increase migration or promote migrant retention.

Following our conceptual framework (Figure 1), food and water security can be enhanced by both migration retention and by migration, depending on the circumstances. In either case, capital accumulation must suffice in order to be able to invest in water and food security. In the case of a potential migrant who remains in the household, if the potential migrant is unemployed or represents surplus labor on a household farm, then livelihoods and food and water security may not be improved. However, if the potential migrant secures off-farm employment or adds benefit to household agricultural labor activities, then livelihoods and food security can be enhanced. Likewise, if a migrant earns insufficiently to send remittances, household livelihoods and food and water security may suffer, but if remittances are sent, they will often be invested first as a priority in food and water security and will contribute to overall livelihood resilience. Similarly, conditions which may retain a potential migrant—a strong labor market, access to health care and education, good public infrastructure, political transparency—also help to improve local, youth, and women’s empowerment. Following Figure 5, vulnerability and resilience to DLDD are embedded within natural and human systems that ultimately produce proximate determinants that either increase migration or promote migrant retention. Some of these proximate factors may include agricultural production, employment, family location, safety, education, health, and political and legal frameworks.

## 3. Conclusions

Addressing the global challenges of desertification, land degradation, and drought (DLDD), and their impacts on achieving sustainable development goals for coupled human-environmental systems is a key component of the 2030 Agenda for Sustainable Development. Addressing this challenge is essential for improving the livelihoods of those most affected by DLDD, and for safeguarding against the most extreme effects of climate change. This paper introduced a conceptual framework for improved monitoring of DLDD in the context of United Nations Convention to Combat Desertification (UNCCD) Strategic Objective 2 (SO2) and its expected impacts: food security and adequate access to water for people in affected areas are improved; the livelihoods of people in affected areas are improved and diversified; local people, especially women and youth, are empowered and participate in decision-making processes in combating DLDD; and migration forced by desertification and land degradation is substantially reduced. While it is critical to develop methods and tools for assessing DLDD, and for understanding the socio-economic conditions of vulnerable communities in affected areas, more work is needed first to better understand the human dimensions of vulnerability in relation to DLDD, especially when attempting to create a globally standardized monitoring approach.

Future research could usefully enhance and critique conceptual frameworks for the implementation of the improved monitoring and evaluation of UNCCD, SDG, and related international frameworks. Future research can also help identify which variables, from which data sources are most appropriate for assessing major international monitoring and evaluation frameworks at different scales. As technology advances, today’s expensive cutting-edge products will be tomorrow’s publicly available data. There is also opportunity to innovate, leveraging extant publicly available datasets. Various spatial statistical methods enable the conversion of data of relatively low spatial resolution to data of higher resolution, with associated location-specific data value probability ranges. Despite ongoing challenges of data scarcity in some remote rural areas within nations, future efforts could expand on what already works. For example, many human health and wellbeing recommended UNCCD and SDG indicators are derived from Demographic and Health surveys (DHS). Yet approximately half the world’s nations have yet to conduct a DHS survey, and those that have could usefully increase the frequency of years in which the data are collected. Similarly, DHS questionnaires are not perfectly uniform. For example, not all countries include the full suite of questions needed to assess women’s empowerment in the household. It would be of great benefit to SO2 and to broader SDG monitoring if these important variables were standardized across all DHS surveys.

There is great promise for the future application potential of this framework. There is a vast literature that could potentially benefit from the integration inherent in the UNCCD Strategic Objectives and the conceptual framework developed in this paper. For example, in relation to the first Strategic Objective, ample research exists on food security (e.g., [21–23]) and access to water (e.g., [24,25]), but few of these (see, e.g., [26,27] for exceptions) which examine these contributions have fully integrated both human and natural systems. Similarly, while there is now a large literature on livelihoods (e.g., [16,28]), important connections to women and youth empowerment and the resilience of both social and ecological systems enhanced are less apparent. Additionally, the scholarly work on women and youth empowerment has grown in recent years (e.g., [29–31]), yet much less research connects women and youth empowerment in relation to combating DLDD. Lastly, there is a whole sub-field in demography for the study of migration, usually focusing on migration determinants often involving international migration (e.g., [32–34]), a much smaller and more recent literature has only begun to connect migration and, conversely, potential migrant retention to resilience to desertification and land degradation (e.g., [35–37]).

There are some points to keep in mind when conducting DLDD case studies, especially in reference to integrating social and remotely sensed data. Ideally, future research can follow an integrative conceptual framework to examine the human dimensions of DLDD applying suitable empirical data inputs. The ideal suite of datasets would be freely available with global coverage and sub-national spatial coverage, while also allowing for disaggregation by gender. For human exposure and livelihoods, freely available datasets include WorldPop’s gridded 100 m global estimation of population density and Demographic and Health Surveys (DHS) data. DHS data are especially useful for examining water access, food security, and gender empowerment. Additional data for examining food security are also available through the Famine and Early Warning Systems Network (FEWSNET) and the National Aeronautics and Space Administration (NASA). For migration data (though with several limitations) WorldPop Migration Flows and Integrated Public Use Microdata Series International (IPUMS-International) can be useful. Lastly, for exploring land and water resources and sustainable management, Intact Forested Landscapes (IFL), NASA Trends in GRACE (for Gravity Recovery and Climate Experiment) and Copernicus and European Space Agency (ESA) land cover data are helpful datasets. Other datasets that may be used to examine one or more human dimensions of DLDD relevant are also available in certain countries at the sub-national level [38]. There are several indicators and indexes that can be derived from freely available data that follow the UNCCD Strategic objectives and the conceptual framework developed here [38]. Food security can be measured by the prevalence of undernourished and the prevalence of severe food insecurity. Livelihoods can be examined using the percent of a population earning below a certain amount, for example the threshold for extreme poverty at $1.90 daily, a gini index of income inequality, and a multidimensional poverty index. Women and youth empowerment can be examined using the gendered disaggregation of the multidimensional poverty index and the women’s empowerment in agriculture index. Lastly, migration can be measured by quantifying the number of people who moved across administrative boundaries, indicating a person’s place of residence 1 year, 5 years, and 10 years ago, respectively.

Subsequent efforts could usefully prioritize building on and improving these efforts both in spatial coverage and in the fidelity and reliability of the spatial modeling techniques. With these and other investments and improvements in data richness, including local case studies, collection and availability, attributions between DLDD and human dynamics promise to be enhanced. These and related efforts will be necessary priorities if country Parties’ SO2 and SDG monitoring capacity development is to match the urgency of the challenges being monitored, and if the research, monitoring, and evaluation is to faithfully reflect the conceptual frameworks introduced here.

## Data Availability

Dara sources used or referenced in this article can be found in more detail at Lopez-Carr, D, K., Mwenda, Mapes, K., Sokolow, S, Linghai Liu, Pricope, N.G. A Review of Publicly Available Geospatial Datasets and Indicators in Support of UNCCD Strategic Objective (SO) 2: To Improve Living Conditions of Populations Affected by Desertification, Land Degradation, and Drought.
